# Advances in CRISPR-Cas9 genome engineering: lessons learned from RNA interference

**DOI:** 10.1093/nar/gkv226

**Published:** 2015-03-23

**Authors:** Rodolphe Barrangou, Amanda Birmingham, Stefan Wiemann, Roderick L. Beijersbergen, Veit Hornung, Anja van Brabant Smith

**Affiliations:** 1 Department of Food, Bioprocessing and Nutrition Sciences, North Carolina State University, Raleigh, NC 27695, USA; 2 Dharmacon, part of GE Healthcare, Lafayette, CO 80026, USA; 3 Division of Molecular Genome Analysis, and Genomic & Proteomics Core Facility, German Cancer Research Center, 69120 Heidelberg, Germany; 4 The Netherlands Cancer Institute, 1066 CX Amsterdam, The Netherlands; 5 Institute of Molecular Medicine, University Hospital, University of Bonn, 53128 Bonn, Germany

## Abstract

The discovery that the machinery of the Clustered Regularly Interspaced Short Palindromic Repeats (CRISPR)-Cas9 bacterial immune system can be re-purposed to easily create deletions, insertions and replacements in the mammalian genome has revolutionized the field of genome engineering and re-invigorated the field of gene therapy. Many parallels have been drawn between the newly discovered CRISPR-Cas9 system and the RNA interference (RNAi) pathway in terms of their utility for understanding and interrogating gene function in mammalian cells. Given this similarity, the CRISPR-Cas9 field stands to benefit immensely from lessons learned during the development of RNAi technology. We examine how the history of RNAi can inform today's challenges in CRISPR-Cas9 genome engineering such as efficiency, specificity, high-throughput screening and delivery for *in vivo* and therapeutic applications.

## INTRODUCTION

From early classical genetic studies to present-day molecular ones, the ability to modulate gene content and expression has been essential to understanding the function of genes within biological pathways and their correlation with disease phenotypes. The discovery of RNAi and its reduction to practice in mammalian cells in the early to mid 2000's made reverse genetics approaches feasible on a genome scale in higher eukaryotes (1). In the last 24 months, another gene modulation technique, Clustered Regularly Interspaced Short Palindromic Repeats (CRISPR)-Cas9 genome engineering (referred to as CRISPR-Cas9), has emerged; in that remarkably brief window, this approach has proven to be a powerful tool for studying individual gene function, performing genome-wide screens, creating disease models and perhaps developing therapeutic agents (2). These lightning advances have largely followed the path blazed by RNAi studies and we argue that further leverage is to be gained by examining relevant successes and failures in the last 14 years of RNAi.

**Figure 1.**
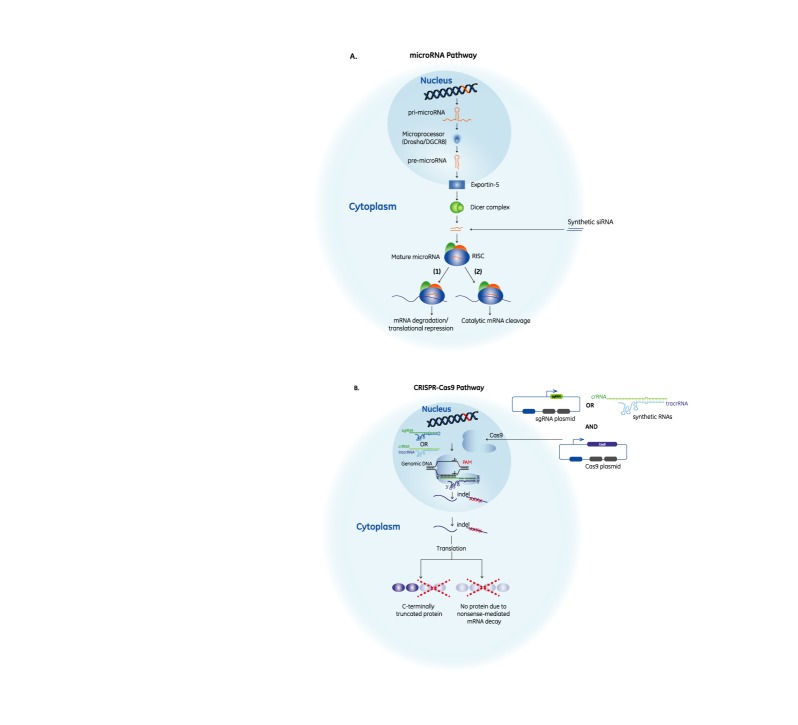
The RNAi and CRISPR-Cas9 pathways in mammalian cells. (**A**) miRNA genes code for primary miRNAs that are processed by the Drosha/DGCR8 complex to generate pre-miRNAs with a hairpin structure. These molecules are exported from the nucleus to the cytoplasm, where they are further processed by Dicer to generate ∼22-nt-long double-stranded mature miRNAs. The RNA duplex associates with an Argonaute (Ago) protein and is then unwound; the strand with a more unstable 5′ end (known as the guide strand) is loaded into Ago to create the RNA-induced silencing complex (RISC) while the unloaded strand is discarded. Depending on the degree of complementarity to their targets, miRNAs cause either transcript cleavage and/or translational repression and mRNA degradation. siRNAs directly mimic mature miRNA duplexes, while shRNAs enter the miRNA pathway at the pre-miRNA hairpin stage and are processed into such duplexes. (**B**) CRISPR-Cas9-mediated genome engineering in mammalian cells requires crRNA, tracrRNA and Cas9. crRNA and tracrRNA can be provided exogenously through a plasmid for expression of a sgRNA, or chemically synthesized crRNA and tracrRNA molecules can be transfected along with a Cas9 expression plasmid. The crRNA and tracrRNA are loaded into Cas9 to form an RNP complex which targets complementary DNA adjacent to the PAM. Using the RuvC and HNH nickases, Cas9 generates a double-stranded break (DSB) that can be either repaired precisely (resulting in no genetic change) or imperfectly repaired to create a mutation (indel) in the targeted gene. There are a myriad of mutations that can be generated; some mutations will have no effect on protein function while others will result in truncations or loss of protein function. Shown are mutations that will induce a frameshift in the coding region of the mRNA (indicated by red X's), resulting in either a truncated, non-functional protein or loss of protein expression due to nonsense-mediated decay of the mRNA.

RNAi and CRISPR-Cas9 have many clear similarities. Indeed, the mechanisms of both use small RNAs with an on-target specificity of ∼18–20 nt. Both methods have been extensively reviewed recently (3–5) so we only highlight their main features here. RNAi operates by piggybacking on the endogenous eukaryotic pathway for microRNA-based gene regulation (Figure 1A). microRNAs (miRNAs) are small, ∼22-nt-long molecules that cause cleavage, degradation and/or translational repression of RNAs with adequate complementarity to them (6). RNAi reagents for research aim to exploit the cleavage pathway using perfect complementarity to their targets to produce robust down-regulation of only the intended target gene. The CRISPR-Cas9 system, on the other hand, originates from the bacterial CRISPR-Cas system, which provides adaptive immunity against invading genetic elements (7). Generally, CRISPR-Cas systems provide DNA-encoded (7), RNA-mediated (8), DNA- (9) or RNA-targeting(10) sequence-specific targeting. Cas9 is the signature protein for Type II CRISPR-Cas systems (11), in which gene editing is mediated by a ribonucleoprotein (RNP) complex consisting of a CRISPR RNA (crRNA) (8) in combination with a *trans*-activating CRISPR RNA (tracrRNA) (12) and a Cas9 nuclease (13–16) that targets complementary DNA flanked by a protospacer-adjacent motif (PAM) (17–19). The molecular machinery from the CRISPR-Cas9 bacterial immune system can be repurposed for genome editing in mammalian cells by introduction of exogenous crRNAs and tracrRNAs or a single guide RNA chimeric molecule (sgRNA) which combines crRNA and tracrRNA sequences, together with the Cas9 endonuclease to create a double-strand break (DSB) in the targeted DNA (16,20–22) (Figure 1B). The DSB is repaired either by non-homologous end joining (NHEJ) or homology-directed repair (HDR) (23). The error-prone NHEJ pathway typically generates small insertions or deletions (indels) that are unpredictable in nature, but frequently cause impactful and inactivating mutations in the targeted sequence; conversely, the HDR pathway is useful for precise insertion of donor DNA into the targeted site.

**Figure 2.**
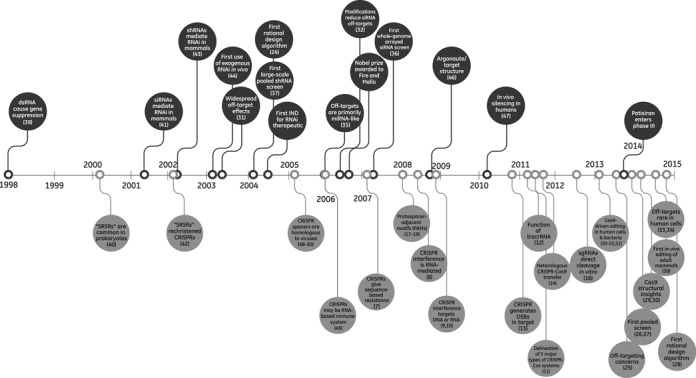
Timeline of milestones for RNAi and CRISPR-Cas9. Milestones in the RNAi field are noted above the line and milestones in the CRISPR-Cas9 field are noted below the line. These milestones have been covered in depth in recent reviews (2,4,52–29).

Both RNAi and CRISPR-Cas9 have experienced significant milestones in their technological development, as highlighted in Figure 2 (7–14,16–22,24–51) (highlighted topics have been detailed in recent reviews (2,4,52–58)). The CRISPR-Cas9 milestones to date have mimicked a compressed version of those for RNAi, underlining the practical benefit of leveraging similarities to this well-trodden research path. While RNAi has already influenced many advances in the CRISPR-Cas9 field, other applications of CRISPR-Cas9 have not yet been attained but will likely continue to be inspired by the corresponding advances in the RNAi field (Table 1). Of particular interest are the potential parallels in efficiency, specificity, screening and *in vivo*/therapeutic applications, which we discuss further below.

**Table 1.** Summary of improvements in the CRISPR-Cas9 field that can be anticipated by corresponding RNAi advances

Milestone	RNAi	CRISPR
IND application	2004	This step is undoubtedly imminent. The drug that was the subject of the first RNAi IND failed clinical trials when its effect was shown to be due to non-RNAi-related mechanisms; especially since CRISPR therapeutics require the delivery not only of a targeting RNA but of exogenous Cas9 (delivered as DNA, mRNA or protein), pharmaceutical developers must avoid allowing history to repeat itself.
Off-target driver identification	2006	Current work is characterizing the nature and extent of the PAM-proximal crRNA ‘seed’. Until it is complete, novel outcomes must be demonstrated using multiple reagents to the same target, as is routinely done for RNAi. Once the crRNA seed is understood, researchers should determine whether it could be leveraged to develop sequence-specific off-target controls such as RNAi's C911 controls.
Off-target-reducing modifications	2006	While effective specificity-enhancing chemical modifications for CRISPR may have to wait until off-target drivers are more fully understood, synthetic crRNAs should be modifiable by precisely the same methods as synthetic siRNAs.
Large-scale arrayed screening	2007	Genome-wide arrayed screens using CRISPR are likely to be more challenging because the percentage of edited cells is typically lower than for RNAi. Nonetheless, CRISPR screening and analysis practices will build on and extend those designed for RNAi screening, just as the latter did with those for small-molecule screening.
*in vivo* use (human)	2010	As CRISPR-driven editing in adult human cells has already been achieved, *in vivo* human use seems inevitable. Efficacious delivery, including that of the exogenous Cas9 protein (or Cas9 mRNA) necessary to make integration-less DNA modifications, is likely to present a significant hurdle. Novel delivery formulations developed in pursuit of RNAi therapeutics will undoubtedly be among those tried first.
Phase III entry	2014	CRISPRa and other dCas9-based approaches raise the hope of addressing conditions untreatable purely via RNAi-like down-regulation while retaining the reversible nature of RNAi. The two modalities might profitably be used in parallel.

## EFFICIENCY

Work performed during the first few years of intensive RNAi investigations demonstrated that, when taking 70–75% reduction in RNA levels as a heuristic threshold for efficiency (59), only a small majority of siRNAs and shRNAs function efficiently (24,60) when guide strand sequences are chosen randomly. This observation led to the development in 2004 of rational design algorithms for siRNA molecules (Figure 2), followed later by similar algorithms for shRNAs. These methods have been able to achieve ∼75% correlation and >80% positive predictive power in identifying functional siRNAs (61) but have been somewhat less effective for shRNAs (62) (perhaps because in most cases, shRNAs produce less knockdown than do siRNAs, likely due to a smaller number of active molecules in each cell). crRNAs also vary widely in efficiency: reports have demonstrated indel (insertion and deletion) creation rates between 5 and 65% (20,25), though the average appears to be between 10 and 40% in un-enriched cell populations. Indeed, a growing amount of evidence suggests a wide range of crRNA efficiency between genes and even between exons of the same gene, yielding some ‘super’ crRNAs that are more functional (26,27). However, such high-functioning crRNAs are likely to make up only a small percentage of those randomly selected for any given gene (28).

Following the RNAi playbook, efforts to develop rational design algorithms for crRNA have already begun: Doench *et al*. (28) assayed thousands of sgRNAs in a functional assay and identified sequence features that predict sgRNA activity. In addition, the CRISPR-Cas9 field has moved quickly to determine relevant structures and details on the separate binding and cleavage processes (29–30,63), thus avoiding a deficit of mechanistic information that impeded early efforts to predict RNAi efficiency. From these early CRISPR-Cas9 studies, it is already understood that the efficiency of the crRNA:tracrRNA:Cas9 RNP complex is determined by PAM-dependent, crRNA-driven Cas9 binding to target DNA as well as crRNA sequence complementarity to the target DNA (particularly in the first PAM-proximal 8–10 nt, the ‘seed’ region of the crRNA). Nonetheless, many details of CRISPR-Cas9 activity remain to be learned. Because an efficient sgRNA or crRNA must not only create a DSB in the sequence of interest, but also create a mutation that results in functional disruption of the resulting protein or non-coding structure, the type of indel as well as its position along the length of the coding sequence are likely to be important; thus, effective rational design efforts for functional knockouts may need to incorporate attributes governing these characteristics as well. Furthermore, more than a decade of efforts in the RNAi field demonstrates that even a well-characterized system is not necessarily easy to predict: crRNA design algorithms, like those for siRNAs and shRNAs, may plateau in their predictive power at a level that still necessitates testing of multiple reagents per target in order to guarantee selection of a functional one.

It is worth noting that efficient gene silencing by an RNAi reagent requires a process of ongoing, active repression; this mechanism may be impaired by gene-specific factors that increase mRNA turnover and/or decrease RNA-induced silencing complex (RISC) turnover. As such, there are some genes for which no RNAi reagents can be found that qualify as effective. In contrast, efficiency of a crRNA depends upon the probability of a one-time event—the editing of a DNA site. Because of this difference, the success of rational design efforts may be less critical to the CRISPR field on an individual gene basis, as it will be widely possible to find an effective crRNA if one is willing to evaluate enough treated cells; highly effective crRNA designs, however, will still be necessary for genome-scale studies, as discussed further below.

## SPECIFICITY AND OFF-TARGET EFFECTS

Perhaps in no other area are the lessons of RNAi as obvious as in that of specificity. While RNAi was originally hailed as exquisitely specific (64), subsequent research has shown that in some circumstances it can trigger non-specific effects and/or sequence-specific off-target effects (65). Many non-specific effects seen with this approach are mediated by the inadvertent activation of pattern recognition receptors (PRRs) of the innate immune system that have evolved to sense the presence of nucleic acids in certain sub-cellular compartments. siRNA length, certain sequence motifs, the absence of 2-nt 3′ overhangs and cell type are important factors for induction of the mammalian interferon response (66–68). Additionally, the general perturbation of cellular or tissue homeostasis by the delivery process itself can also trigger unwanted responses (most likely secondary to innate immune damage-sensing pathways) such as the wide-spread alteration of gene expression caused by cationic lipids, especially when used at high concentrations (69). Such non-specific effects associated with delivery will still exist for CRISPR-Cas9 but can likely be overcome by minimizing lipid concentration as is now routinely done in RNAi studies. Similarly, the introduction of chemical modifications into the backbone of an siRNA duplex (e.g. 2′-O-methyl ribosyl) can block the recognition of RNA molecules by PRRs (66,70–71), so such modifications may also address innate immune system recognition caused by synthetic crRNAs. Researchers would do well to investigate whether additional effects may result, potentially in a cell-line or cell-type dependent manner, as a response to creation of DSBs or the abundant expression of Cas9 or an sgRNA molecule (such as that seen when strong shRNA expression outcompetes that of endogenous miRNAs, leading to the breakdown of cellular regulation (72)). These types of non-specific off-target effects have already been reported with other genome engineering techniques (e.g. zinc finger nucleases (73)) but the ease-of-use and simplicity of the CRISPR-Cas9 system should allow researchers to address these types of questions fully in the near future.

RNAi can also produce sequence-specific off-target effects, which were initially described in early 2003 (31), but whose potential impact was not fully appreciated until well after the method had become a widely used research and screening technique (e.g. (74)). Cleavage-based off-targeting, which occurs when RISC encounters an unintended transcript target with perfect or near-perfect complementarity to its guide strand, can induce knockdown equivalent to that of intended target down-regulation and was originally hypothesized to be the main cause of sequence-specific off-target effects. It took several years to determine that these effects were in fact primarily caused by RNAi reagents acting in a ‘miRNA-like’ fashion, down-regulating unintended targets by small (usually <2-fold) amounts primarily through seed-based interactions with the 3′ UTR of those unintended targets. Because miRNA-like off-targeting is generally seed-based and all transcripts contain matches to a variety of 6–8-base motifs, such off-targeting can affect tens to hundreds of transcripts. Furthermore, if the RNAi reagent contains a seed mimicking that of an endogenous miRNA, the off-targeting may affect the pathway or family of targets evolutionarily selected for regulation by that miRNA. It is not possible to design RNAi reagents that do not contain seed regions found in the transcriptome's 3′ UTRs and the non-seed factors that conclusively determine whether or not a seed-matched transcript is in fact off-targeted have not yet been identified. Both rational design and chemical modifications such as 2′ O-methyl ribosyl substitutions can mitigate seed-based off-target effects (32), but without a full solution, specificity remains a well-known pain point for RNAi users.

Inspired by these concerns, an initial evaluation of the off-target potential of CRISPR-Cas9 was published within months of the technique's debut and work to refine these early findings has continued apace. Studies have revealed some sequence flexibility, and tolerance for mismatches and bulges, that have generated concerns about specificity and sequence-directed off-target cleavage (25,75–79). Several variations of CRISPR-Cas9 have been developed to address specificity including paired nickases (77), short sgRNAs (76) and Cas9 fused to FokI (80), and the rapid advances in understanding CRISPR-Cas9 mechanism and structure are likely to further fuel such developments. Recent papers, however, have uncovered very few to no off-target mutations that can be attributed to CRISPR-Cas9 (33,34) and conclude that clonal artifacts that derive from isolating CRISPR-Cas9-edited cells may be a larger concern. This apparent discrepancy between prediction and reality, while encouraging, highlights a treacherous pitfall in studying off-target gene editing: to date the primary approach has been to predict putative off-target sites and then search for editing at those sites, but this approach risks falling prey to the ‘streetlight effect’, in which one searches only where it is easy to look. The RNAi field learned this lesson painfully: early off-target prediction efforts focused on strong overall complementarity as a determinant and thus largely failed to identify genes that were actually off-targeted due to short, seed-based complementary (35). Unless the CRISPR-Cas9 field learns from RNAi's mistakes, it is in danger of repeating the same very one, especially as CRISPR-Cas9 specificity has recently also been shown to depend on an as-yet-not-fully-defined seed region (20,81). At this stage, computational predictions of putative off-target gene-editing sites are at best questionable guesses and thus cannot be depended upon in assessing the full effect of off-targeting. Unfortunately, while RNAi specificity studies are limited to the transcriptome, analysis of CRISPR-Cas9 specificity requires identifying effects that may occur anywhere in the genome, generally through next-generation sequencing. This process is costly and depends upon non-trivial data analysis and processing, so it remains unclear whether the field has the will to commit to this work. Until such time as a less biased understanding of CRISPR-Cas9 off-targeting emerges, researchers are advised to emulate the best-practice of RNAi by using multiple crRNAs or sgRNAs in order to show redundancy of phenotype by multiple reagents targeting the same gene, thus ensuring that the phenotype is due to on-target effects rather than off-target effects.

## GENOME-SCALE SCREENING TOOLS

Interest in genome-scale CRISPR-Cas9-based screening has blossomed, with some pooled screening resources already available (26,27) and arrayed ones likely to emerge in the near future. Because genome editing screens will also be affected by a large number of the factors that make RNAi screens more challenging than small-molecule ones (82), practitioners would do well to study the hard-won victories in this field since the first published whole-genome synthetic lethal screen for sensitization to paclitaxel (36) (Figure 2) before diving into these costly experiments.

Of particular importance is evaluating whether the lower efficiencies seen using CRISPR-Cas9 are sufficient to generate a desired phenotype in the screening assay—that is, determining whether the phenotype is detectable in the targeted cell population. In this regard, two factors are of special concern: the ploidy of the gene locus of interest (as tumor cell lines are often aneuploid) and the likelihood of disrupting the reading frame by the induced mutation (since +3 or −3 indels would not serve this purpose). Taking these factors into account, the chance of obtaining a high percentage of cells that have a functional knockout in a bulk cell culture is relatively low under typical screening conditions. Consequently, it is unlikely that traditional arrayed loss-of-signal screens such as those common in RNAi will be widely feasible in bulk-transfected cells using CRISPR-Cas9. Nevertheless, the CRISPR-Cas9 technology may have an advantage in screens for which a complete knockout is required to uncover a phenotype (for example, when targeting kinases where a residual expression of 10% is sufficient for activity). The use of analysis techniques that examine effects at the single-cell level (using, for example, high-content microscopy or fluorescence-activated cell sorting-based read-outs) could also be informative, as it has been for gene silencing screens. A more novel possibility is leveraging HDR to develop systems in which CRISPR-Cas9-mediated DSBs are repaired using an exogenous template harboring a marker or resistance gene that can later be used to select cells with a functional, inactivating, recombination event (83).

Homologous recombination could similarly be used to validate RNAi screening hits by introducing variants in the transcript that do not alter the protein but create mismatches with the siRNA or shRNA reagents such that target knockdown no longer occurs, providing a fast method to distinguish on-target and off-target effects. More simply, gene editing can be used to knock out a putative hit to determine if the knockout results in a similar phenotype to that obtained with knockdown during the RNAi screen (84). Although (as discussed further below) knockout phenotypes may differ from knockdown phenotypes (so that a negative result may not necessarily indicate a false positive), confirmation of the knockdown phenotype with a CRISPR-Cas9 knockout would be a strong indication of a true positive. Conversely, RNAi reagents may be an effective validation strategy for CRISPR-Cas9-based screens and the numerous other effective approaches optimized for RNAi screens, such as confirmation of assay phenotype by multiple independent reagents and the use of additional related assays (58), will prove directly applicable to genome editing validation.

The development and application of RNAi-based pooled screening approaches have greatly enhanced the field of functional genomics screening in mammalian cells: the ability to quantify in large populations the relative abundance of individual cells, each carrying a gene-specific gene-modifying reagent, allows for different screening models such as identification of genotype-specific essential genes, synthetic lethal genes or genes involved in resistance to specific drugs (85). Crucial requirements for this approach are the availability of reagents with high efficiency and specificity, the presence of tractable markers for integrated constructs and the availability of large collections of gene-perturbing reagents in retroviral or lentiviral vectors. In contrast to the current lack of arrayed screening resources for CRISPR-Cas9, large, genome-scale collections of sgRNAs that fulfill these criteria are available alongside analogous shRNA collections. Numerous examples of both pooled RNAi screens and pooled CRISPR-Cas9 screens have been published (26–27,86–90,37).

CRISPR-Cas9 pooled screens share with their RNAi-based cousins the necessity of inactivating gene activity at single-copy integration of the shRNA- or sgRNA-encoding expression cassette. This proves a difficulty for both technologies, as efficiency of shRNA-mediated knockdown varies for different platforms but never reaches complete knockdown for all vectors targeting a specific gene (91,92) and the observed frequency of CRISPR-Cas9-based gene inactivation using single-copy sgRNAs shows great variability among the different studies, on average not reaching frequencies higher than 50% (20,25). RNAi screeners have demonstrated that such limited efficiency is more challenging for screening models aimed at the identification of lethal genes, synthetic lethal genes or response enhancers than for enrichment experiments like resistance screens or positive selection of a phenotype such as expression of a cell surface marker. To combat this limitation, libraries for CRISPR-Cas9 pooled screening are already including multiple independent reagents for each gene. As shown by shRNA-based pooled screens, this approach insures against false negatives due to individual inefficient reagents and provides a means of corroborating each reagent's result, thereby reducing false positives.

False-positive off-target effects in both RNAi and CRISPR-Cas9 pooled libraries will also be mitigated by rational design, as discussed above. Further, recently developed C911 seed match controls (93) can be implemented in large-scale shRNA screening collections as internal off-target matched controls; such a technique would be highly desirable for CRISPR-Cas9 screening but its feasibility will depend on further understanding of the relevant specificity mechanisms. So far, the levels of off-target effects in pooled screens using CRISPR-Cas9-based gene editing remains unclear, although results from the comparison of shRNA and CRISPR-Cas9 screens in the same screening model (for genes whose loss confer resistance to vemurafenib, a BRAF protein kinase inhibitor) in A375 melanoma cells support a low frequency of off-target effects (26) in the CRISPR-Cas9 system. With regards to false negatives, one would expect that a considerable advantage of the CRISPR-Cas9 technology over shRNA-mediated knockdown would be increased strength of phenotype due to the ability to completely abolish expression of the targeted gene. As a result, one would predict improved recovery of genes involved in the phenotype of interest, and indeed, it has been reported that the recovery rate for essential genes is higher for CRISPR-Cas9 than for shRNA (94). However, the aforementioned genome-wide CRISPR-Cas9 screen for cellular resistance to vemurafenib identified a limited number of hits compared to a similar screen with a genome-wide shRNA collection (26). It may be that CRISPR-Cas9-based screens are unable to identify genes that are lethal upon complete loss, but are associated with the desired phenotype when knocked down by 70–90%. An example of such a gene is SOX10, which causes a slow-growth phenotype upon knockdown; this phenotype is associated with resistance to vemurafenib (95). CRISPR-Cas9 screeners should take this potential bias into account when analyzing their screening results.

## *IN VIVO* STUDIES

Following the footsteps of RNAi, CRISPR-Cas9 has quickly advanced beyond studies in cell lines and primary cell cultures to *in vivo* studies aimed at everything from examination of the biology of particular genes and disease phenotypes to development of potential therapeutic agents. Notably, however, this technology provides significant advances in the creation of animal models for mechanistic studies that RNAi, given its transient and partial nature, cannot offer. Focusing on *in vivo* studies in the mouse, Wang *et al*. (96) demonstrated that CRISPR-Cas9 can be introduced into embryonic stem cells in a multiplex fashion to create animals carrying multiple specific mutations in several genes in a manner requiring only one generation, thereby dramatically decreasing the time required to generate transgenic animal models. Additional studies have used CRISPR-Cas9 to create mouse models of various cancers by mutating a combination of tumor suppressor genes and oncogenes in the livers of wild-type mice (97) or in mouse hematopoietic stem cells (98), eliminating the need for time-consuming creation and crossbreeding of genetically engineered mouse strains. The modification of the mouse genome using CRISPR-Cas9 technology is not limited to gene mutations: large chromosomal deletions, inversions and translocations can be produced by using multiple sgRNAs (99–101).

The final goal of much *in vivo* work is the development of therapeutic tools. In spite of challenges regarding delivery and non-specific effects (including those that caused the first RNAi-based therapeutic candidate by OPKO Health to fail phase III clinical trials in 2009), considerable efforts and investments continue in the pursuit of RNA-targeting therapeutics. More than 30 clinical trials are currently in progress or completed on indications from pachyonychia congenita to high cholesterol (102,103). Recently, advances in non-viral delivery systems have been made with the development of lipopeptide nanoparticles that offer the opportunity to treat disease via *in vivo* delivery to endothelial cells or hepatocytes (104,105). Given this enduring interest in gene-modulation-based drugs, it seems certain that CRISPR-Cas9-based treatments will shortly enter the therapeutics pipeline; recent proof-of-principle studies (Table 2) point to likely indications (106–115). Gene-editing therapeutics may enjoy a smoother road than gene-silencing-based ones since they have no requirement for continuous delivery of siRNAs or continuous expression of integrated shRNAs. As a consequence, gene editing can be done without leaving a footprint in the genome other than the corrected DNA sequence. While gene-editing therapeutics may have the advantage of not requiring continuous delivery or expression of RNAs, RNAi has the advantage of using endogenous eukaryotic protein machinery such that only small RNAs must be delivered or expressed. In contrast, CRISPR-Cas9-based therapeutics requires delivery of the Cas9 gene, mRNA or protein (which is quite large if using the canonical protein from *Streptococcus* *pyogenes*). Hence, delivery—the bête noire of RNAi therapeutics—may continue to be a therapeutic challenge. Recent work, however, has demonstrated effective *in vivo* editing via lipid-mediated delivery of CRISPR-Cas9 protein complexed with an sgRNA (116). Nonetheless, RNAi's long and bumpy road to the clinic should temper hopes for an immediate CRISPR-Cas9-driven revolution in drug development.

**Table 2.** Summary of publications demonstrating use of CRISPR-Cas9 for targeting disease

Disease	Summary
Cataracts	Rescue of a dominant mutation in the Crygc gene that causes cataracts (108).
Cystic fibrosis	Correction of the CFTR locus by homologous recombination in cultured intestinal stem cells from patients with cystic fibrosis (109).
β-thalassemia	Correction of the human hemoglobin beta (HBB) gene in induced pluripotent stem cells from β-thalassemia patients using CRISPR-Cas9 and the piggyback transposon (106).
HPV-associated cervical cancer	Targeting of promoters of human papillomavirus oncogenes; inhibited tumorigenesis (110).
Hereditary tyrosinemia type I	Correction of the Fah mutation in hepatocytes of a mouse model of hereditary tyrosinemia (107).
HIV	Generation of homozygous CCR5 deletion mutations in iPSCs; proposed approach toward a functional cure of HIV-1 infection (111). Targeting of LTR sequences in the HIV-1 genome; inactivated viral gene expression and replication in latently infected cells and prevented new HIV-1 infection (112).
Malaria	High (50–100%) gene disruption of the *Plasmodium falciporum* genome. Potential to generate transgenic parasites to prevent malaria (113).
Duchenne Muscular Dystrophy (DMD)	2–100% correction of the DMD mutation in the dystrophin gene in the germ line of a mouse model of DMD (114).
Herpesviridae infection	Targeting of genomes of latent herpesviridae viral infections; suggests use as an antiviral treatment in human cells (115).

Intellectual property (IP) portfolios determine who reaps the rewards of any therapeutic technology, and given the abundant promise of CRISPR-Cas9 systems, it is perhaps unsurprising that the IP landscape surrounding them has already proven to be complex, competitive and rapidly evolving (117). Like RNAi, CRISPR-Cas9 is sure to be the source of vigorous IP disputes and negotiations in the coming years. Notably, however, the first patents containing claims fundamental to the system have already been granted at a pace that is unprecedented. We expect the CRISPR-Cas9 IP portfolio will not fully emerge for a few more years, but clearly there is significant commercial interest and technological value in establishing ownership of foundational IP in this area.

## FUNDAMENTAL DIFFERENCES

RNAi has demonstrated tremendous value as a functional genomics tool, especially with the technological advances described above that enhance efficiency and decrease off-target effects (118). Likewise, CRISPR-Cas9 has already proven to be a valuable tool for functional genomics studies. Although we have highlighted many points on which the RNAi field can offer pertinent guidance for the effective development and exploitation of CRISPR-Cas9, it is important to remember the fundamental differences that underlie these techniques (Table 3). These contrasts must be considered when selecting the most appropriate method for studying a particular gene or genome.

**Table 3.** Summary of differences between RNAi and CRISPR-Cas9

Feature	RNAi	CRISPR-Cas9
Mode of action	Knocks gene down at mRNA or non-coding RNA level.	Modifies gene (via knockout/knockin) at the genomic DNA level.
	Utilizes the endogenous mammalian microRNA machinery.	Can be used to facilitate site-specific modifications of sequences, including the introduction of single nucleotide variants (SNVs) and the insertion of tags.
	Typically occurs in the cytoplasm.	Derives from the exogenous CRISPR-Cas Type II adaptive immune system in bacteria.
		Occurs in the nucleus.
Duration of effect	Gives transient effect (siRNA) to long-term effect (shRNA).	Causes permanent and heritable change in the genome.
Efficiency	Typically induces >75% knockdown. Generates phenotypic effect typically detectable in a cell population.	Typically induces 10–40% editing per allele.
	Does not require clonal isolation.	Generates phenotypic effect that may not be detectable in a cell population.
		Usually requires clonal isolation.
Design of functional components	Can employ reagents targeted all along transcript.	Can employ only reagents with targets adjacent to PAM and (for gene knockout) in a critical exon.

### Molecular consequences

One such fundamental difference between the two is the molecular consequences of their actions. RNAi results in knockdown at the RNA level while CRISPR-Cas9 causes a change in the DNA of the genome; as a corollary, RNAi happens predominantly in the cytoplasm, while CRISPR-Cas9 acts in the nucleus. These contrasts highlight the differing applicability of the techniques: for example, circRNAs (119,120) that differ from their linear counterparts by splice order in the final transcript can be interrogated by RNAi but not CRISPR-Cas9, while intron functionality can be investigated by CRISPR-Cas9 but not RNAi. For more prosaic targets of interest, in some cases the resulting phenotype associated with either knockdown or knockout may be similar but in others there may be significant differences that result from repression of gene expression compared to a complete null genotype. Although CRISPR-Cas9-based approaches for drug target identification have been developed (121), repression of gene expression may better model a potential drug's means of activity and thus be more relevant for drug discovery efforts.

### Duration of effect

Because of differences in their mode of action, CRISPR-Cas9 and RNAi also differ in their duration of effect. siRNA knockdown is typically transient (lasting 2–7 days), while genome engineering with CRISPR-Cas9 induces a permanent effect that, if all alleles are affected, sustainably removes gene function and activity. shRNA knockdown can be either short- or long-term depending on whether the shRNA is continuously expressed, providing some middle-ground; shRNA activity can also be turned on and off with inducible vectors (122,123) although some leakage can occur even in the off state, depending on the inducible system. Inducible or transient systems will also likely be necessary for studying essential genes via CRISPR-Cas9, but unlike in shRNA systems, there will be no ability to toggle back once knockout is induced. For non-essential targets, a preference for transience or permanence will be determined by the researcher's goal; since most drug treatments have a transient effect, RNAi will likely mimic them more effectively than CRISPR-Cas9 knockouts.

### Modulation of non-coding genes

Most protein-coding genes will be easily down-modulated by either RNAi or CRISPR-Cas9. For permanent disruption of protein-coding genes using CRISPR-Cas9, frameshift mutations in a critical coding exon (i.e. an early protein-coding exon that is used by all relevant transcript variants) must occur, while RNAi reagents can be targeted essentially anywhere within the transcript. However, knockdown or knockout of non-coding RNAs is more nuanced. The study of small non-coding genes, particularly, is complicated for both RNAi and CRISPR-Cas9 by the limited design space for targeting the non-coding gene without affecting nearby genes. This concern is particularly relevant for the silencing of miRNAs, many of which are encoded within introns of protein-coding host genes. CRISPR-Cas9-mediated knockout of miRNAs may have the potential to be more efficient than antagomir knockdown as there is just one design of an antagomir per miRNA (i.e. the reverse complement of the mature miRNA) while a miRNA gene can be targeted by CRISPR-Cas9 at several sites such as the promoter, hairpin, etc. Targeting of promoters can also be achieved using a catalytically inactive Cas9 in combination with sgRNA (CRISPRi) to precisely interfere with the transcriptional machinery (124). On the other hand, antagomirs can be designed to target specifically either the 5-prime or 3-prime arm of the mature miRNA while CRISPR-Cas9 knockout will target both. CRISPR-Cas9 also opens the door to novel techniques for verifying miRNA targeting, as it has been successfully applied to generate mutant miRNA binding sites in target genes (125).

A further consideration is that many non-coding gene products, both small and large, localize and act only or mostly in the nucleus (e.g. MALAT-1 (126), XIST (127)). Since the occurrence of RNAi-induced knockdown of nuclear RNAs has been under debate for quite some time (128), RNAi against nuclear non-coding RNAs is possible but not certain while knockout of ncRNA genes using CRISPR-Cas9 should have similar efficiency to that against protein-coding genes. On the other hand, RNAi cleavage efficiently degrades ncRNAs; CRISPR-Cas9-based insertion or deletion of a few bases may not be sufficient to significantly compromise the activity of ncRNAs, as they are not vulnerable to frameshifting and may act through overall structure that is difficult to disrupt. One possible solution to this problem is the targeted deletion of larger genomic regions. Indeed, the inactivation of a particular lncRNA *in* *vivo* using this strategy has been reported (129).

It is worth noting that the CRISPR-Cas9 system has been further developed to modulate gene expression at the transcriptional level, not just at the genomic DNA level. Specifically, fusion proteins using a catalytically deactivated Cas9 (dCas9) can be used to direct either transcriptional activator or repressor domains to a gene of interest using sgRNAs directed against promoter regions through techniques known as CRISPRa and CRISPRi (124,130–134). Enhancements to the system have been developed as well as genome-scale resources for targeting genes at the transcriptional level for both CRISPRi and CRISPRa (135,136). The CRISPRi technology now has similar advantages to RNAi as outlined above—that is, transience, reversibility and partial suppression. CRISPRa, on the other hand, provides a unique capability for activating gene expression globally because although RNA activation (RNAa) using short RNA duplexes and components of the RNAi machinery has been reported in mammalian cells, its mechanism is not well understood and it has not proven to be universal (reviewed in(137)).

### CRISPR-specific concerns

For permanent disruption of either coding or non-coding genes using CRISPR-Cas9, the ploidy of the gene locus of interest is an important consideration because it is known that in many tumor cell lines, as well as in plants, there is considerable aneuploidy (138) and polyploidy (139). CRISPR-Cas9-engineered clones with one, two (or, if present, more) or all modified alleles will be ideal tools for studying dominant versus recessive phenotypes, gene dosage, haploinsufficiency and related phenomena; the editing of three alleles in hexaploid bread wheat has already been achieved and used to assess the relevance of these genes for plant phenotypes (140). RNAi cannot be used to study these types of phenomena because gene and protein dosages cannot be consistently controlled using RNAi.

Of course, while CRISPR-Cas9 has so far been applied most widely for gene disruption via NHEJ, it has the potential to create precise genomic additions through HDR. The targeted insertion of mutations into endogenous gene copies is a unique feature of the CRISPR-Cas9 system compared to RNAi, allowing direct testing of the functional effects of such mutations *in vitro* (141) and *in vivo* (38). For example, CRISPR-Cas9 has been used to insert tags into endogenous loci to produce labeled proteins under the control of the endogenous regulation machinery, allowing functional testing of these proteins at physiological expression levels *in vivo* (142). Previously, these types of studies could only be performed in an indirect manner, requiring, for dominant effects, testing of ectopically expressed mutant constructs in isogenic cell lines (143), for example, or, for recessive conditions, the generation of knockout animals and ectopic expression of wild-type and mutant constructs. Recently, the generation of tumor-specific chromosomal translocations using CRISPR-Cas9 technology has also been achieved, mimicking genetic alterations observed in many tumor genomes (100). Furthermore, as noted above, the reversal of mutations in globulin genes with thalassemia mutations in iPS cells (106) has opened the possibility of human gene therapy for certain diseases. Insertion as well as reversal of mutations is currently limited by the design of crRNAs or sgRNAs requiring a PAM sequence to be present in the right context. New Cas9 enzymes are on the horizon, likely having different sequence requirements that should help to overcome this issue. When fully realized as a research tool, HDR-based mutagenesis will provide a capability entirely distinct from RNAi's down-regulation.

### Ease of use

In terms of ease of use, transfections of siRNAs and shRNAs are fast and simple whereas transductions with shRNAs require production of lentiviral or retroviral particles but are still relatively straight-forward. RNAi, therefore, remains a time- and cost-effective technology, while gene engineering with CRISPR-Cas9 depends not only on transfection or transduction but also selection, verification of induced variation and clonal expansion of engineered cells or organisms. In this context, it should be noted that CRISPR-Cas9 engineering of primary cell types (most importantly post-mitotic cells) has so far only been reported using adenoviral vectors instead of the commonly applied lentiviral backbone (144). For these reasons, the CRISPR-Cas9 technology requires more time and effort input than RNAi. Consequently, the choice of technology is determined by the biology, scope and effort required.

## DISCUSSION

In the past two years, the CRISPR-Cas9 genome editing technology has burst onto the scene and advanced by leaps and bounds. In much of this development, it has already leveraged the history of the more mature field of RNAi and we highlight additional lessons to be learned from RNAi's successes and failures relating to efficiency, specificity, screening and *in vivo* applications. However, the fact that CRISPR-Cas9 is not an endogenous mammalian system provides the opportunity for innovative protein evolution studies that are not possible with RNAi. Given this, we anticipate that the CRISPR-Cas9 field will expand beyond the canonical *S. pyogenes* SpyCas9 in combination with the NGG PAM that has been the focus of virtually all mammalian applications to date. Indeed, other Cas9 proteins are being increasingly characterized (145) with their respective PAMs (of various sizes and sequences) in order to expand targeting specificity. Further, protein engineering studies for the rational design of Cas9 nucleases with improved specificity and enhanced targeting efficiencies are certainly underway, employing the structural information discovered for the *S. pyogenes* (SpyCas) and *Actinomyces naelslundii* (AnaCas9) Cas9 proteins (29). In the very near future, CRISPR-Cas9 has the opportunity to both complement and extend the RNAi method, avoiding the pitfalls RNAi has experienced while building on its successes. With both of these technologies, researchers certainly have an impressive toolbox for elucidating gene function and advancing science and medicine.
